# Supplementary figures and images for: Honokiol decreases alpha-synuclein mRNA levels and reveals novel targets for modulating alpha-synuclein expression
Source: Front Aging Neurosci. 2023 Aug 10;15:1179086. doi: 10.3389/fnagi.2023.1179086 (PMC10449643; doi:10.3389/fnagi.2023.1179086)

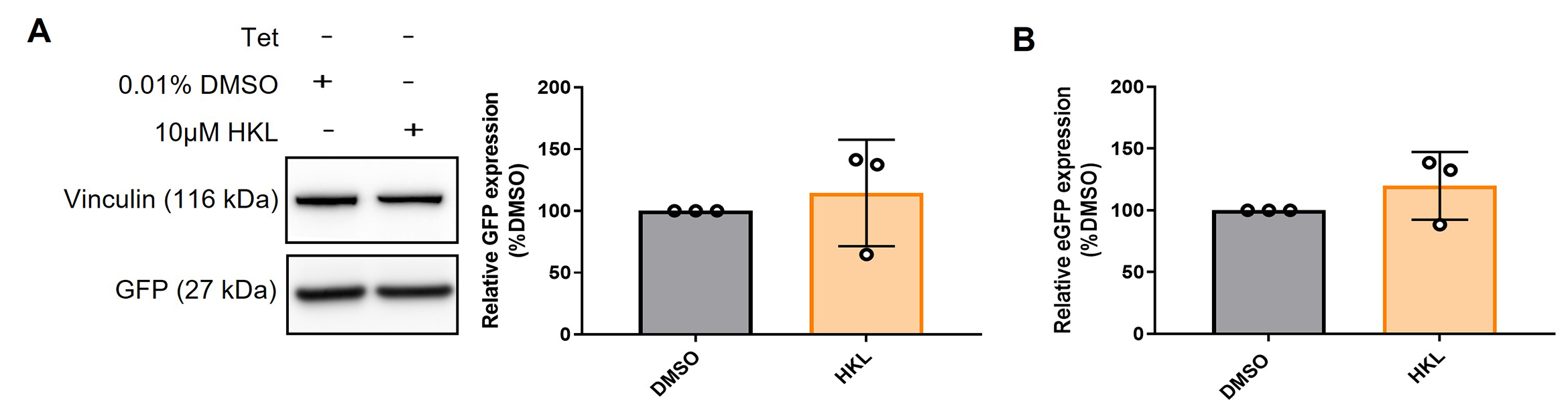

Supplement: Supplementary Figure 1 — HKL does not affect GFP expression in H4 cells stably overexpressing wt-αsyn. Western blot and quantification of GFP expression following treatment with 10 μM HKL [n = 3 biological replicates/treatment, t(4) = 0.58, p = 0.59] (A). Effects of HKL treatment in eGFP mRNA levels [n = 3 biological replicates/treatment, t (4)1.25, p = 0.28] (B). Data are analyzed with unpaired Student’s t-test and are represented as mean ± SEM. [file Image_1.JPEG]

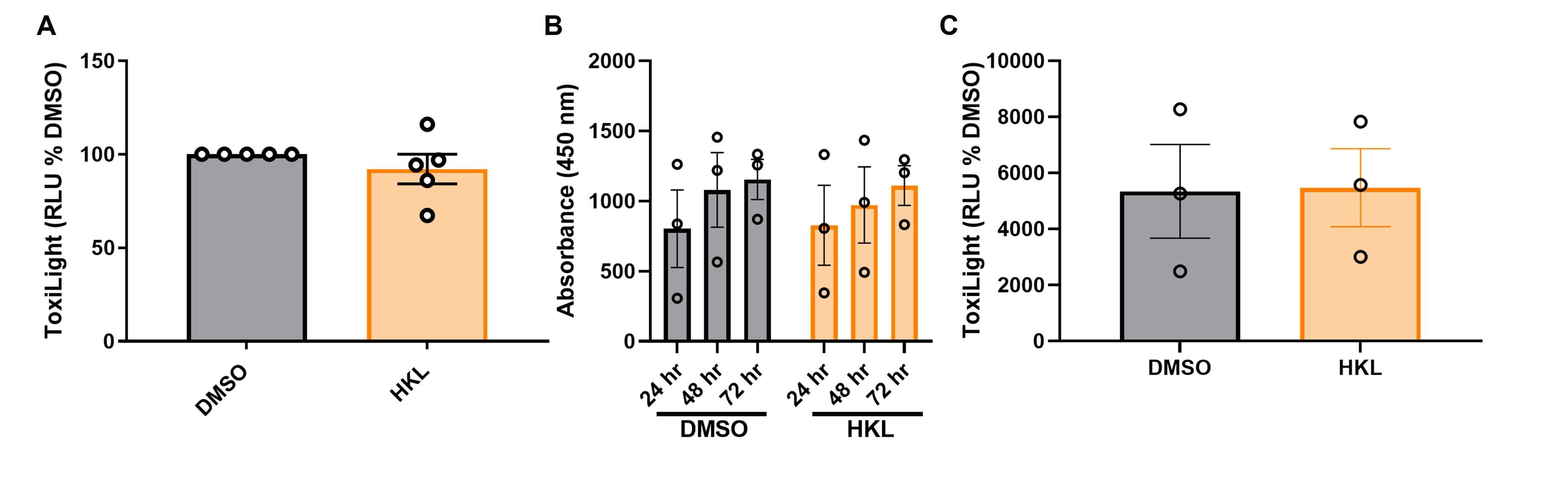

Supplement: Supplementary Figure 2 — HKL does not induce toxicity nor affect cell proliferation. The Toxilight assay was performed in H4 wt-αsyn cells after 72 h treatment with 10 μM HKL [n = 5 biological replicates, t (8) = 0.10, p = 0.35] (A). Proliferation WST-1 assay in H4 wt-αsyn cells assessed over 72 h of HKL treatment (n = 3 biological replicates, repeated measures two-way ANOVA, time F (1.13, 9.03) p < 0.05, treatment F (3, 8) = 0.35 p = 0.35, time X treatment F (6, 16) = 0.17 p = 0.98) (B). Toxicity of 72 h treatment with 10 μM HKL on mouse primary cortical neuron was evaluated in the Toxilight assay [n = 3 biological replicates, t (4) = 0.06, p = 0.96] (C). Data are analyzed with unpaired Student’s t-test and are represented as mean ± SEM. [file Image_2.JPEG]

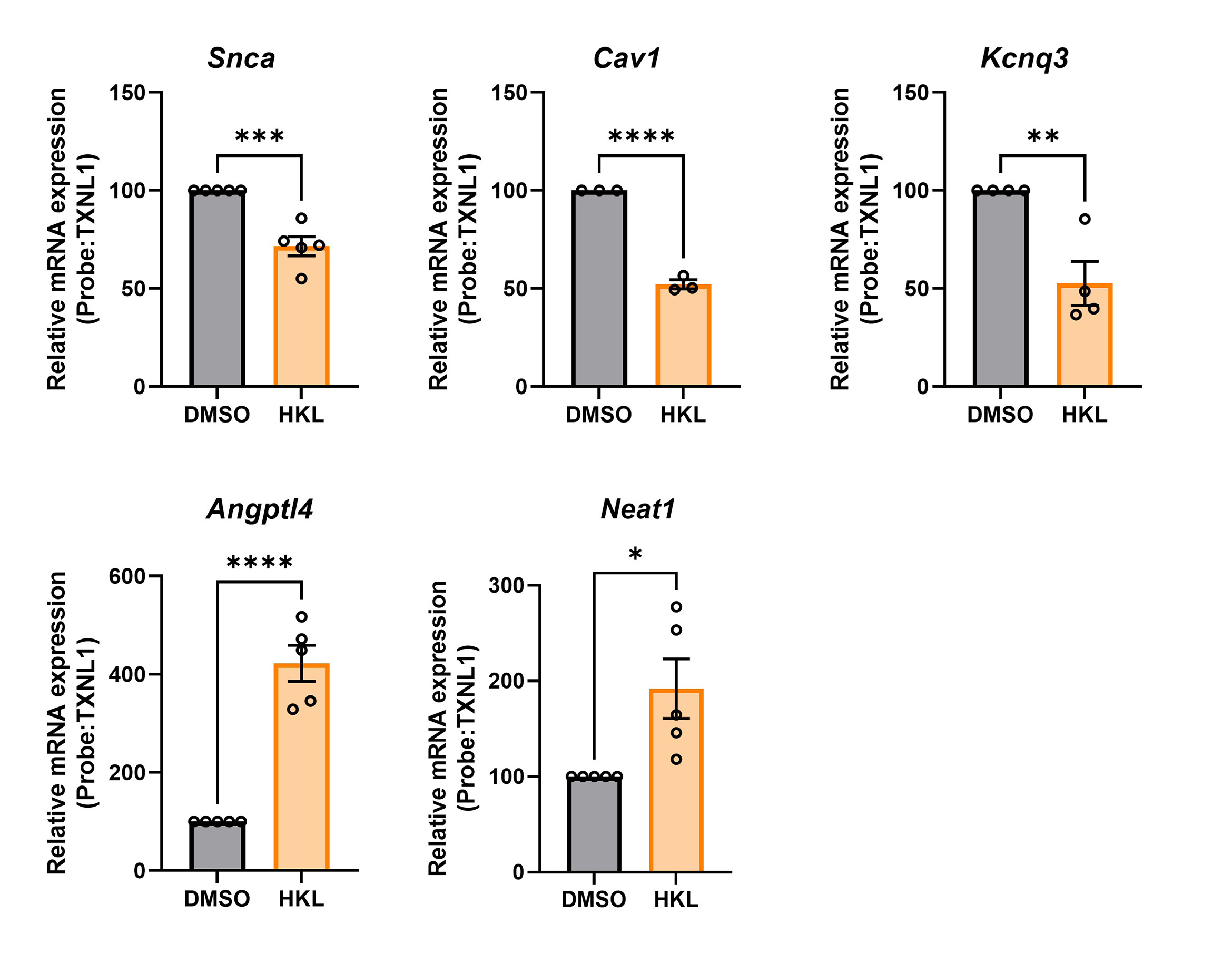

Supplement: Supplementary Figure 3 — Validation of DEGs in mouse primary cortical neurons. Of the top 25 differentially expressed genes (DEGs) identified with bulk RNA sequencing in mouse primary cortical neurons, the following were validated to confirm the effect of HKL in these cells. Data were analyzed with Student’s t-test and are represented as mean ± S.E.M. ****Cav1 - t (4) = 21.12, p = < 0.0001, **Kcnq3 - t (6) = 4.21, p = 0.0056, ****Angplt4 - t (8) = 8.82, p = < 0.001, *Neat1, t (8) = 2.95, p = 0.0184, ***Snca t (8) = 5.79, p = 0.0004. [file Image_3.JPEG]
