# Supplementary material for: Honokiol decreases alpha-synuclein mRNA levels and reveals novel targets for modulating alpha-synuclein expression
Source: Front Aging Neurosci. 2023 Aug 10;15:1179086. doi: 10.3389/fnagi.2023.1179086 (PMC10449643; doi:10.3389/fnagi.2023.1179086)
Supplement: Supplementary file 4 [file Table_1.docx]

Table 1. List of primary and secondary antibodies.

| **Primary Antibodies** | | | |
| --- | --- | --- | --- |
| **Antibody #** | **Manufacturer** | **Dilution** | **Method** |
| Vinculin #V9131 | Sigma | 1:5,000 | Western Blot |
| GAPDH #AP7873a | Abgent | 1:5,000 | Western Blot |
| Actin #A5060 | Sigma | 1:10,000 | Western Blot |
| GFP #ab6556 | Abcam | 1:2,000 | Western Blot |
| αsyn #610787 | BD | 1:1,000-1:2,000 | Western Blot |
| αsyn (4B12) #807802 | BioLegend | 1:1,000-1:2,000 | Western Blot |
| αtubulin #T9026 | Sigma | 1:1,000 | ICC |
| αsyn (MJF) #ab138501 | Abcam | 1:1000 | ICC |
| SNCA #571241 | ACD | Plate dependent | RNAscope |
| DAPI #320851 | ACD | Plate dependent | RNAscope |
| **Secondary Antibodies** | | | |
| HRP Anti-ms #626520 | Invitrogen | 1:2,000 | Western Blot |
| HRP Anti-rb #656120 | Invitrogen | 1:2,000 | Western Blot |
| Alexa 488 | ACD | 1:1,000 | RNAscope |
| Alexa 568 # A11004 | Invitrogen | 1:1,000 | ICC |
| Alexa 647 # A21235 | Invitrogen | 1:1,000 | ICC |
