# Supplementary material for: Honokiol decreases alpha-synuclein mRNA levels and reveals novel targets for modulating alpha-synuclein expression
Source: Front Aging Neurosci. 2023 Aug 10;15:1179086. doi: 10.3389/fnagi.2023.1179086 (PMC10449643; doi:10.3389/fnagi.2023.1179086)
Supplement: Supplementary file 5 [file Table_2.DOCX]

Table 2. List of qPCR Taqman probes.

| **Taqman Probe** | **Product #** |
| --- | --- |
| Txnl1 | Mm00497442_m1 |
| Snca | Mm01188700_m1 |
| Angptl4 | Mm00480431_m1 |
| Neat1 | Mm05873557_s1 |
| Cav1 | Mm00483057_m1 |
| Kcnq3 | Mm00548884_m1 |
| HPRT1 | Hs02800695_m1 |
| SNCA | Hs00240906_m1 |
